# Targeted 11C–choline PET-CT/TRUS software fusion-guided prostate biopsy in men with persistently elevated PSA and negative mpMRI after previous negative biopsy

**DOI:** 10.1186/s41824-017-0011-1

**Published:** 2017-11-01

**Authors:** Massimo Lazzeri, Egesta Lopci, Giovanni Lughezzani, Piergiuseppe Colombo, Paolo Casale, Rodolfo Hurle, Alberto Saita, Lorenzo Leonardi, Giuliana Lista, Roberto Peschechera, Luisa Pasini, Marcello Rodari, Silvia Zandegiacomo, Alessio Benetti, Pasquale Cardone, Federica Mrakic, Luca Balzarini, Arturo Chiti, Giorgio Guazzoni, Nicolò Maria Buffi

**Affiliations:** 1 Department of Nuclear-Medicine, Humanitas Clinical and Research Hospital, Milan, Rozzano Italy; 2 Department of Urology, Humanitas Clinical and Research Hospital, via Manzoni 56, 20089 Milan, Rozzano Italy; 3 Department of Pathology, Humanitas Clinical and Research Hospital, Milan, Rozzano Italy; 4 Department of Radiology, Humanitas Clinical and Research Hospital, Milan, Rozzano Italy; 5 Humanitas University, Milan, Rozzano Italy

## Abstract

**Background:**

We evaluated the feasibility and accuracy of 11C–choline PET-CT/TRUS fusion-guided prostate biopsy in men with persistently elevated PSA and negative mpMRI or contraindication to MRI, after previous negative biopsy. Clinical data were part of a prospective on-going observational clinical study: “Diagnostic accuracy of target mpMRI/US fusion biopsy in patients with suspected prostate cancer after initial negative biopsy”. Patients with a negative biopsy and negative mpMRI (PI-RADS v.2 < 3) or absolute contraindications to MRI and persistently elevated PSA, were included. All patients underwent 11C–choline PET with dedicated acquisition of the pelvis and PET-CT/TRUS-guided prostate biopsy by Bio-Jet™ fusion system (D&K Technologies, Germany). The primary endpoint was to assess the accuracy of 11C–choline PET-CT to determine the presence and the topographical distribution of PCa.

**Results:**

Overall, 15 patients (median age 71 yrs. ± 8.89; tPSA 13.5 ng/ml ± 4.3) were analysed. Fourteen had a positive PET scan, which revealed 30 lesions. PCa was detected in 7/15 patients (46.7%) and four patients presented a clinically significant PCa: GS > 6. Over 58 cores, 25 (43.1%) were positive. No statistically significant difference in terms of mean and median values for SUVmax and SUVratio between benign and malignant lesions was found. PCa lesions with GS 3 + 3 (*n* = 3) showed a median SUVmax and SUVratio of 4.01 and 1.46, compared to 5.45 and 1.57, respectively for lesions with GS >6 (*n* = 4).

**Conclusion:**

Software PET-CT/TRUS fusion-guided target biopsy could be a diagnostic alternative in patients with a suspected primary PCa and negative mpMRI, but its specificity appeared low.

## Introduction

mpMRI has become the preferred method for detecting prostate cancer (PCa) foci after initial negative random biopsy (Schoots et al., 2015). In order to improve the use of mpMRI within urological and radiological community, the Prostate Imaging Reporting and Data System (PI-RADS) v. 1 and, recently, v. 2 score were established (Hamoen et al., 2015; Weinreb et al., 2016). Currently mpMRI is incorporated into national and international guidelines (https://www.nice.org.uk/guidance; http://www.uroweb.org) for PCa detection in men with prior negative biopsy findings.

Although the negative predictive value (NPV) of mpMRI is around 95%, some tumours may not be detected. In a preliminary study, 122 men treated with radical prostatectomy, preoperative mpMRI failed to detect GS ≥ 7 tumours and tumours >1 cm diameter in 28% of cases (Le et al., 2015). Filson C. et al. confirmed such data. They found that systematic biopsies revealed clinically significant PCa in 16% of men with no suspicion of prostate cancer at mpMRI biopsy (Filson et al., 2016). If biopsy is indicated on clinical grounds, a negative mpMRI, which could miss a third of significant prostate cancer (Futterer et al., 2015), should not preclude it. Furthermore, MRI may have absolute or relative contraindications and therefore, there is an unmet need to find alternative imaging for target biopsy.

Molecular imaging with PET is one of the most promising tools for the investigation of PCa (Scher et al., 2007; Hernández-Argüello et al., 2016). PET-guided prostate biopsy implementation could be not only plausible, but also mandatory in some subjects with contraindications to MRI or high clinical probability of prostate cancer, associated to negative MRI. Although radio-labelled 11C–choline PET studies have provided equivocal results, because of some overlap with benign prostate hyperplasia and prostatitis (Scher et al., 2007), the sensitivity for tumour foci in prostate gland can be high enough to complement MRI findings in the final diagnosis (Hernández-Argüello et al., 2016).

The aim of the current study was to assess the potential clinical impact of 11C–choline PET-CT/TRUS software fusion-guided prostate biopsy in men with persistently elevated PSA after negative biopsy and negative mpMRI.

## Material & methods

Clinical data were part of a prospective on-going observational clinical study: “Diagnostic accuracy of target mpMRI/US fusion biopsy in patients with suspected prostate cancer after initial negative biopsy” approved by our Institutional Review Board (IRB) and Ethical Committee. Written informed consent was obtained from all individual participants included in the study. Patients with persistently elevated PSA, with or without ASAP and/or HG-PIN and negative DRE, after at least one negative biopsy (at least 12 cores for each biopsy course) and a negative mpMRI (PI-RADS v.2 < 3) or contraindications to MRI were included.

### 11C–choline PET/CT imaging technique

**Fig. 1:**
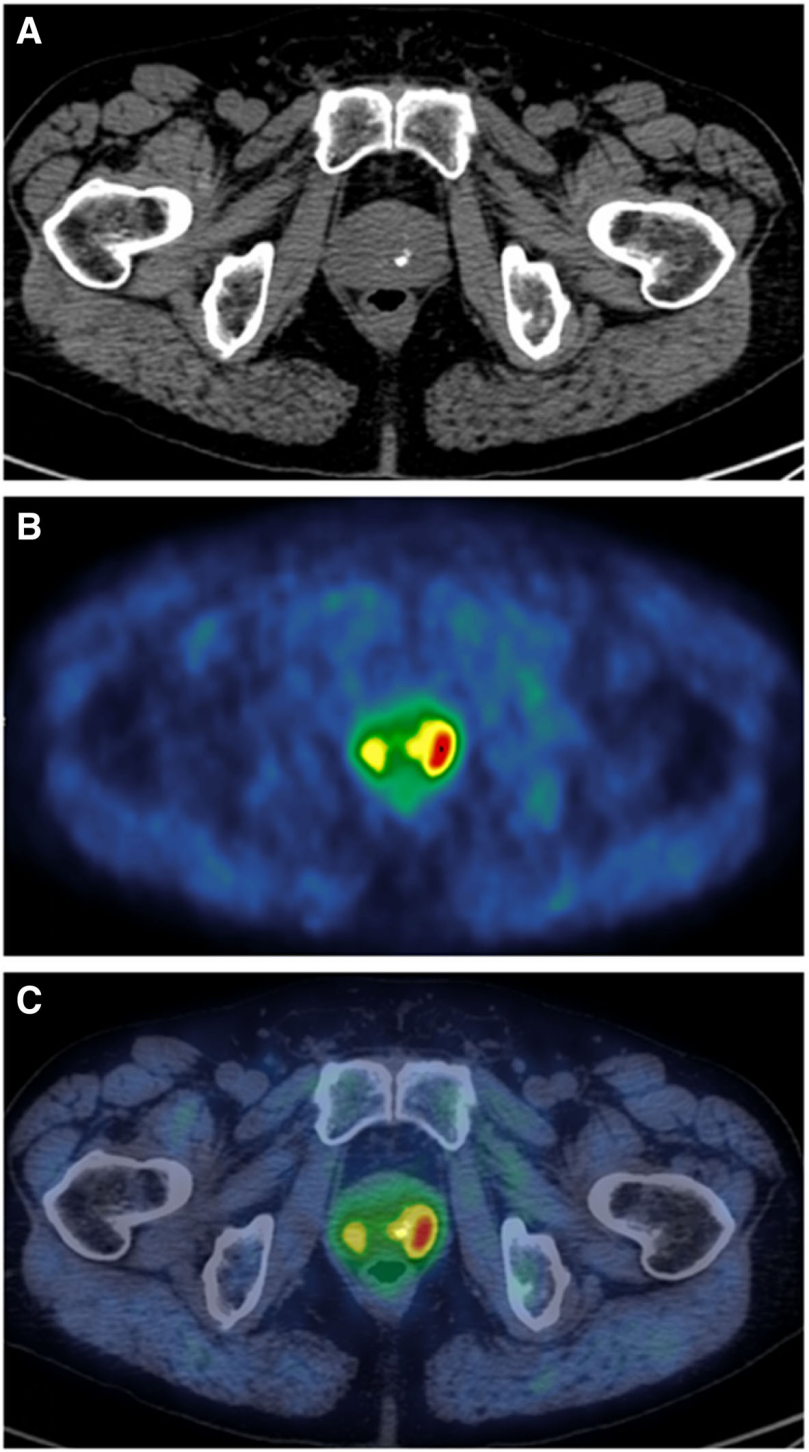
Focal uptake detected on the periphery of the left prostatic lobe. **a** CT scan; **b** 11C-choline PET scan; **c** 11C–choline PET/CT fused scan

The radiopharmaceutical 11C–choline was synthesized using a General Electric TracerLab FXc module as previously described by (Pascali et al., 2000), and administered in a total amount of 250–400 MBq. Ten minutes after tracer administration, body axial images from the mid-thigh to the skull base were obtained with an integrated PET/CT Discovery 690, GE Healthcare. Low-dose CT images were acquired using an automated dose modulation (maximal 140 mA, 140 kVp), 64 × 3,75 mm collimation, 3.75 mm slice thickness, 0.5 s rotation time, and pitch 0,984:1. Emission images were obtained with a 3.0 min acquisition for bed position in the pelvis and 1.5 min for the remaining total body acquisition (axial FOV 1150 mm, matrix size 256 × 256) and subsequently reconstructed using an iterative algorithm (VUE Point FX, 3 iterations, 24 subsets). Reconstructed images of the pelvis were obtained separately and displayed for reading as sagittal, axial and coronal views on OsiriX MD Imaging workstation (Fig. 1). A correlation between PET/CT prostate volume and ultrasound prostate volume using the BK Medical, Analogic Ultrasound Group, Pro Focus, Transducer 8818, 6/9 MHz, was also performed. The ellipsoid formula was used (PV = p/6 x [length (cm)] x [width (cm)] x [high (cm)]). PET was considered positive when focal uptake of 11C–choline was superior to the background activity.

### Software PET/TRUS fusion-guided target biopsy technique

For PET/TRUS fusion-guided prostate biopsy, the Bio-Jet™ fusion system and software (D&K Technologies, Barum, Germany) were used. Shoji et al. described the technical data and usage of this system and Tewes et al. showed that Bio-Jet™ fusion system and software facilitate diagnosis of PCa with high sensitivity and specificity (Shoji et al., 2015; Tewes et al., 2015).

PET/CT DICOM images were uploaded and prostate profile and ROIs were manually contoured. During the biopsy session the prostate and ROIs PET/CT derived image contours were fused in real time with the TRUS image stack and ROIs were targeted (Fig. 1). Biopsies, transrectal or transperineal according to the site of lesions, were performed with patients in dorsal lithotomy position, under antibiotic prophylaxis and local anaesthesia, using a 3D triplane transrectal ultrasound system (BK Medical, Analogic Ultrasound Group, Pro Focus, Transducer 8818, 6/9 MHz). Biopsy cores were numbered according to ROI number and topography. Specimens were processed and evaluated by a genitourinary pathologist. Foci of tumour were quantified (percentage and mean length in millimetres) and graded according to the 2005 consensus conference on Gleason grading of prostatic carcinoma of the International Society of Urological Pathology (Epstein et al., 2005). A second, independent, pathologist (PC), expert in urological pathologies, reviewed all the samples.

The primary endpoint was to assess the accuracy of 11C–choline PET/CT to determine the presence and the topographical distribution of the tumour foci.

Data were complemented by descriptive statistical analysis. For continuous data, differences between groups were compared by the *T*-test or the Wilcoxon-test, when appropriate. Pearson’s correlation coefficient was used for compare US and CT generated prostate volume. Statistical significance was set at *p* ≤ 0.05 for each evaluation. Study analyses were performed on MedCalc statistical software (MedCalc Software bvba © 1993–2014, http://medcalc.org/

## Results

Of 298 consecutive patients enrolled from April 2015 to September 2016, 15 patients (median age 71 yrs. ± 8.89; tPSA 13.5 ng/ml ± 4.3) fulfilled the inclusion criteria: negative mpMRI (PI-RADS v.2 < 3). Twelve mpMRI were performed by 3-Tesla device and 3 by 1.5 Tesla machine with endo-rectal coil.

### 11C–choline PET

PET scan documented the presence of 30 foci of increased radiopharmaceutical uptake, analysed using regions of interest (ROIs) in 14 out of 15 patients; the patients with negative PET/CT (# 5) did not receive any further imaging assessment or biopsy procedure. The ROI median size was 12 mm (range 8–22). Seven ROIs were in the peripheral zone, while 23 were located in the transition zone. The semi-quantitative uptake values of ROIs detected on 11C–choline PET were as follows: median SUVmax 5.17 (range 3.3–8.3), median background SUV 2.97 (range 2–5.54), and SUVratio to background 1.6 (range 1.21–2.51) (Table 1).

**Table 1:** PET-CT outcome and pathological characteristics of biopsies for all the patients (TZ = transition zone; PZ = peripheral zone)

Case	Site	SUV_max_	SUV_background_	Core(+)/Total core	Diagnosis	Gleason score	Tumor volume mm(+)/mm tot (%)
1	Right lobe (TZ)	4,93	3	0/2	BPH	–	–
	Left lobe (PZ)	7,18	2,86	0/2	Prostatitis	–	–
	Left lobe (TZ)	6,4	2,9	0/1	BPH	–	–
2	Right lobe (TZ)	5,79	3,66	6/6	Ca	5 + 4	80/84 (95%)
3	Right lobe (PZ)	5,7	4,09	0/1	BPH	–	–
	Left lobe (TZ)	5,7	2,94	0/1	BPH	–	–
	Right lobe (TZ)	5,83	4,36	0/1	BPH	–	–
4	Right lobe (TZ)	4,6	3,48	0/2	BPH	–	–
	Left lobe (TZ)	5,07	3,3	0/3	BPH	–	–
5	Left lobe (TZ)	1,9	1,63	–	NO BX	–	–
6	Right lobe (TZ)	7,02	3,43	6/7	Ca	4 + 3	31/83 (37,3%)
7	Left lobe (TZ)	4,01	2,75	3/3	Ca	3 + 3	1,5/31 (4,3%)
	Left lobe (TZ)	4,54	2,75	0/1	BPH	–	–
8	Right lobe (TZ)	3,71	2,61	2/3	Ca	3 + 3	9/24 (37%)
	Left lobe (TZ)	3,38	2,61	0/4	BPH	–	–
9	Right lobe (PZ)	3.74	2.4	6/8	Ca	3 + 5	90,5/114 (79,4%)
10	Right lobe (TZ)	5,52	3,92	0/3	BPH	–	–
	Left lobe (TZ)	6,31	2,97	0/3	BPH	–	–
11	Right lobe (TZ)	4.64	2.97	0/2	BPH	–	–
	Left lobe (TZ)	4.81	2.97	0/2	BPH	–	–
	Left lobe (TZ)	5.22	2.97	0/2	BPH	–	–
12	Left lobe (TZ)	5.11	3.35	1/2	Ca	3 + 4	4/24 (16,7%)
13	Right lobe (PZ)	6.17	2.92	0/2	BPH	–	–
	Left lobe (TZ)	6.51	2.92	0/6	BPH	–	–
14	Left lobe (TZ)	6.68	5.54	0/1	BPH	–	–
	Left lobe (PZ)	7.91	5.54	0/2	BPH	–	–
	Right lobe (TZ)	8.33	5.54	0/5	BPH	–	–
15	Right lobe (TZ)	4	2	0/4	BPH	–	–
	Right lobe (PZ)	4.18	2	0/4	BPH	–	–
	Left lobe (TZ)	4.33	2	1/4	Ca	3 + 3	1,1/52 (2,1%)
	Left lobe (PZ)	3.32	2	0/4	BPH	–	–

### Biopsy

**Fig. 2:**
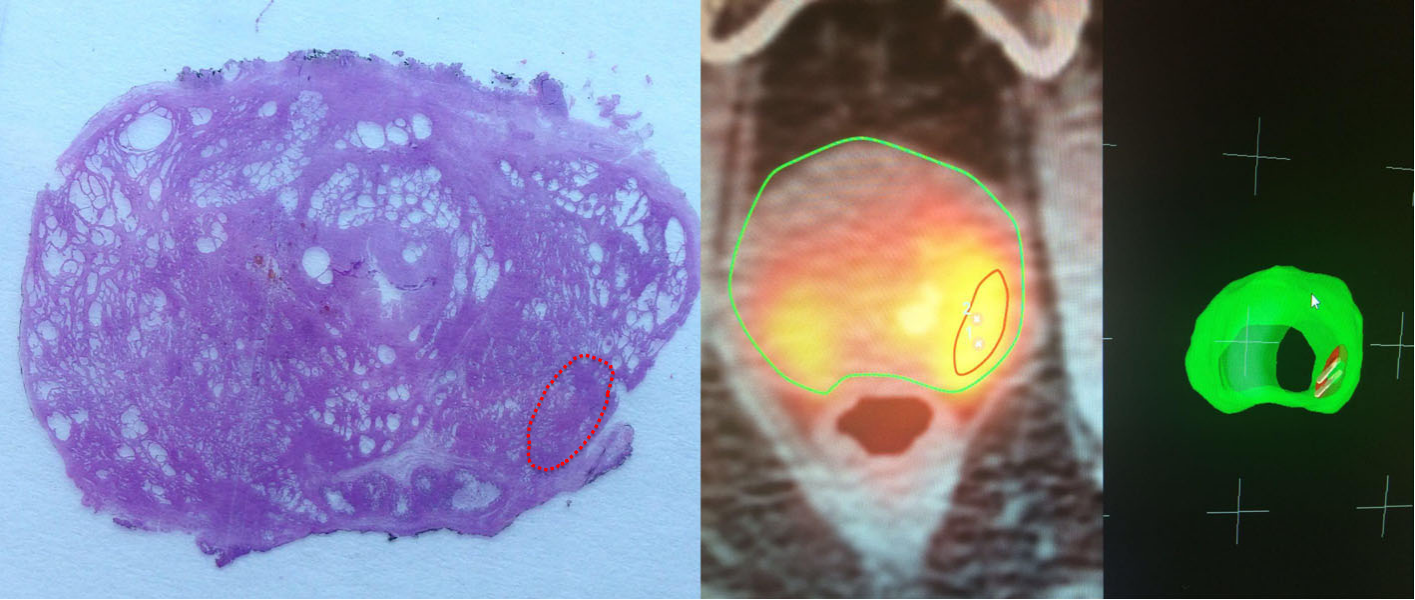
Left: Whole gland pathological examination. Red dot line indicates the PCa index lesion. Centre: Axial PET-CT/TRUS fusion imaging. Green line is the contour of whole gland and red line the ROI. Right: 3D imaging with biopsy samples (red cylinder)

No significant complications due to biopsy were recorded. PCa was detected in 7/15 (46.7%). Over 58 cores, 25 (43.1%) were positive. In one patient PCa was located in the peripheral zone (Figs. 1 and 2), while 6 patients had PCa in transition zones. Four patients harboured a significant PCa: Gleason score ≥ 7. Three patients had a Gleason score 6. One patient showed a granulomatous prostatitis and the others a benign prostatic hyperplasia. A spatial correlation between PET-CT and topography of index lesions was found in a patient who underwent radical prostatectomy (Fig. 2).

### Pathology

Mean involvement in the positive core patients was higher in GS= > 7 than GS = 6 patients (95%, 79,4%, 37,3%, and 16,7%, compared to 37%, 4,3%, and 2,1% respectively). The mean extension (length in mm) of prostatic adeno-carcinoma was higher in GS= > 7 patients than GS = 6 patients (mm13, 5, 15, and 4 versus mm6.5, 4.5, and 1.1 respectively) (Table 1). We found no statistically significant difference in terms of mean and median values for SUVmax and SUVratio between benign and malignant lesions (Table 2). Patients with BPH presented a median SUVmax and SUVratio of 4.93 and 1.65, respectively; inflamed prostatic gland (granulomatous prostatitis) showed a median SUVmax and SUVratio of 5.7 and 1.47, respectively; PCa lesions with GS 3 + 3 showed a median SUVmax and SUVratio of 4.01 and 1.46, compared to 5.45 and 1.57, respectively for lesions with GS >6.

**Table 2:** Values of semi quantitative uptake according to biopsy findings

SUVmax	BPH	Prostatitis	GS3 + 3	GS > 6	*p* value	Ratio	BPH	Prostatitis	GS3 + 3	GS > 6	*p* value
Mean	5.42	5.74	3.91	5.42	0.225	Mean	1.74	1.7	1.64	1.68	0.969
Median	4.93	5.7	4.01	5.45	0.162	Median	1.65	1.47	1.46	1.57	0.725

## Discussion

MpMRI has become the preferred method for detecting cancer-suspicious regions in the prostate, especially in men with a previous negative biopsy, and with a few caveats, it showed to be capable of identifying many serious cancers (Cash et al. 2016). Currently there is robust evidence that MRI is substantially more accurate than US guidance, as the latter usually fails to reveal a valid target within the prostate. mpMRI information was used according three different schemes: cognitive-, in bore- and software assisted TRUS fusion-biopsy; the latter is currently adopted in many urological departments. However, the standardization and validation of mpMRI, including quality assurance in acquisition, reading, and reporting, operator skill and experience in drawing ROIs under a grey scale and performing the biopsy along with the non-negligible rate of false negative, leave room to identify other imaging strategies.

PET imaging has been recently considered one of the most promising approaches for PCa detection. The prerequisite for the diagnosis of primary PCa in PET is the presence of a high tumour-to-background ratio and the correlation of imaging findings with whole gland pathological samples. Although controversies exist on the accuracy of 11-Choline PET for primary detection of PCa (Souvatzoglou et al., 2011), 11C–choline PET scanning could be considered an alternative in all those men with persistent risk of harbouring PCa after a negative mpMRI. The idea of PET guidance for biopsies in men with elevated PSA-levels and a negative mpMRI is not new. In 2008 a study was published in which patients with persistent elevated PSA and previous negative prostate biopsy, were investigated with 18F–choline PET/CT to delineate PCa and guide a repeat biopsy (Igerc et al., 2008). However, they used a cognitive technique and not a software fusion biopsy. In a recent randomized prospective trial, Taverna et al. confirmed the poor accuracy of 3-T mpMRI associated with systematic cognitive biopsies (Taverna et al., 2016). One of the strengths of our approach is that all the patients underwent software assisted fusion biopsy.

Patients with an aggressive PCa (GS > 6) presented with a high tracer uptake, median SUVmax 5.45 and SUVratio to background 1.57, although no statistically significant difference was found. Piert et al. reported that uptake of 11C–choline was significantly increased in high GS lesions (≥4 + 3), with a MIB-1/Ki-67 labeling index ≥5% (*p* = 0.01) in comparison to lesions with GS 3 + 4 or lower (Piert et al., 2009). In this context, we found a high sensitivity, but a low specificity of 11C–choline PET/TAC at software assisted target biopsy.

We must acknowledge some limitations of our study. First of all, the sample size remains small. We did not perform a consensus re-reading of the mpMRI by different prostate imaging experts. The difference of SUVratio between positive and negative patients was not significant and no speculation about a potential SUVratio cut-off between clinically significant vs. indolent PCa can be done. The combination of mpMRI and PET would be superior to both modalities alone, but although it could improve the specificity, it would significantly increase costs.

In conclusion, notwithstanding we showed a “proof of concept” for software PET-CT/TRUS fusion-guided target biopsy, we found a low specificity for 11C–choline PET/CT although a good correlation with pathological outcome. Further studies with a larger population or new tracers such as PSMA could be investigated in order to improve the accuracy of the PET/CT fusion software assisted biopsy in a specific sub set of patients with a suspected primary PCa.

## Abbreviations

ASAP: Atypical Small Acinar Proliferation
DRE: Digital Rectal Examination
HG-PIN: High Grade Prostatic Intraepithelial Neoplasia
mpMRI: multi parametric Magnetic Resonance Imaging
PET/CT: Positron Emission Tomography/Computed Tomography
TRUS: Trans Urethral Ultra Sound

## References

[CR1] Cash H, Maxeiner A, Stephan C (2016). The detection of significant prostate cancer is correlated with the Prostate Imaging Reporting and Data System (PI-RADS) in MRI/Transrectal ultrasound fusion biopsy. World J Urol.

[CR2] Epstein JI, Allsbrook WC, Amin MB, Egevad LL, ISUP Grading Committee (2005). The 2005 International Society of Urological Pathology (ISUP) Consensus Conference on Gleason Grading of Prostatic Carcinoma. Am J Surg Pathol.

[CR3] Filson CP, Natarajan S, Margolis DJ, Huang J, Lieu P, Dorey FJ (2016). Prostate cancer detection with magnetic resonance-ultrasound fusion biopsy: The role of systematic and targeted biopsies. Cancer.

[CR4] Futterer JJ, Briganti A, De Visschere P (2015). Can clinically significant prostate cancer be detected with multiparametric magnetic resonance imaging?.

[CR5] Hamoen EH, de Rooij M, Witjes JA, Barentsz JO, Rovers MM (2015). Use of the Prostate Imaging Reporting and Data System (PI-RADS) for Prostate Cancer Detection with Multiparametric Magnetic Resonance Imaging: A Diagnostic Meta-analysis. Eur Urol.

[CR6] Hernández-Argüello M, Quiceno H, Pascual I (2016). Index lesion characterization by (11) C-Choline PET/CT and Apparent Diffusion Coefficient parameters at 3 Tesla MRI in primary prostate carcinoma. Prostate.

[CR7] Igerc S, Kohlfürst H, Gallowitsch J, Matschnig S, Kresnik E, Gomez-Segovia I, Lind P (2008). The value of 18F–Choline PET/CT in patients with elevated PSA-level and negative prostate needle biopsy for localisation of prostate cancer. Eur J Nucl Med Mol Imaging.

[CR8] Le JD, Tan N, Shkolyar E (2015). Multifocality and prostate cancer detection by multiparametric magnetic resonance imaging: correlation with whole-mount histopathology. Eur Urol.

[CR9] Pascali C, Bogni A, Itawa R, Cambie M, Bombardieri E (2000). [11C]Methylation on a C18 Sep-Pak cartridge: a convenient way to produce [N-methyl-11C]choline. J Labelled Comp Rad.

[CR10] Piert M, Park H, Khan A (2009). Detection of aggressive primary prostate cancer with 11C-choline PET/CT using multimodality fusion techniques. J Nucl Med.

[CR11] Scher B, Seitz M, Albinger W (2007). Value of 11C-choline PET and PET/CT in patients with suspected prostate cancer. Eur J Nucl Med Mol Imaging.

[CR12] Schoots IG, Roobol MJ, Nieboer D, Bangma CH, Steyerberg EW, Hunink MG (2015). Magnetic resonance imaging-targeted biopsy may enhance the diagnostic accuracy of significant prostate cancer detection compared to standard transrectal ultrasound-guided biopsy. Eur Urol.

[CR13] Shoji S, Hiraiwa S, Endo J (2015). Manually controlled targeted prostate biopsy with real-time fusion imaging of multiparametric magnetic resonance imaging and Transrectal ultrasound: an early experience. Int J Urol.

[CR14] Souvatzoglou M, Weirich G, Schwarzenboeck S (2011). The sensitivity of [11C]choline PET/CT to localize prostate cancer depends on the tumor configuration. Clin Cancer Res.

[CR15] Taverna G, Bozzini G, Grizzi F (2016). Endorectal multiparametric 3-tesla magnetic resonance imaging associated with systematic cognitive biopsies does not increase prostate cancer detection rate: a randomized prospective trial. World J Urol.

[CR16] Tewes S, Hueper K, Hartung D (2015). Targeted MRI/TRUS fusion-guided biopsy in men with previous prostate biopsies using a novel registration software and multiparametric MRI PI-RADS scores: first results. World J Urol.

[CR17] Weinreb JC, Barentsz JO, Choyke PL (2016). PI-RADS Prostate Imaging - Reporting and Data System: 2015, Version 2. Eur Urol.

